# Verification of rebuild-up effect on superficial cardiac lesion of ventricular tachycardia using 3-D printed phantom in volumetric-modulated arc therapy planning

**DOI:** 10.1038/s41598-022-05149-3

**Published:** 2022-02-10

**Authors:** Shiwon Roh, Jun-Bong Shin, Yong-Ki Bae, Jungmin Kim, Semie Hong, Jeong-Woo Lee

**Affiliations:** 1 Department of Convergent Medical Physics, Graduate School of Engineering, Konkuk University, 120-1 Neungdong-ro, Gwangjin-gu, Seoul, 05030 Republic of Korea; 2 Department of Radiation Oncology, Kangwon National University Hospital, Chuncheon, South Korea; 3 Department of Radiation Oncology, Konkuk University Medical Center, Seoul, South Korea; 4 Department of Health and Safety Convergence Science, Korea University, Seoul, South Korea

**Keywords:** Radiotherapy, Medical research, Oncology

## Abstract

The aim of the study was to evaluate dose distributions on the superficial cardiac lesion surrounded by low-density lungs. Volumetric modulated arc therapy (VMAT) technique was applied to optimize the dose distribution using the anisotropic analytic algorithm (AAA) and Acuros XB algorithm (AXB) using the 3-D printed cardiac phantom. We used four full and half arcs with 6-MV and 15-MV photons to investigate the rebuild-up effect near the planning target volume (PTV). Depending on the calculation algorithm (AAA vs. AXB) for full arcs plans, V_95_ of PTV differed by 27% for 6-MV and 29% for 15-MV, and D_95_ for 6-MV and 15-MV shows 24% and 30%, respectively. The maximum doses in the AXB plans on PTV were 5.1% higher than those in AAA plans at 6-MV, and 3.8% higher at 15-MV. In addition, half arcs treatment plans showed a very similar tendency with full arcs plans. Film dosimetry showed significant differences from the planned results in the AAA plans. Particularly, the dose mismatch occurred between the cardiac PTV and the left lung interface. In the case of 6-MV plans calculated by AAA, the maximum dose increased from 4.1 to 7.7% in the PTV. Furthermore, it showed that 50% of the width of dose profiles was reduced by 1.3 cm in the 6-MV plan. Conversely, in the case of the plans using the AXB algorithm, the maximum dose increased by 2.0–5.0%. In contrast to the AAA algorithm, the dose patterns at the interface demonstrated a good agreement with the plans. Dose fluctuation on the interface between superficial cardiac lesions and low-density lungs can lead to an error in the estimation of accurate dose delivery for the case of VT SBRT.

## Introduction

According to the GLOBOCAN 2020 database developed by the International Agency for Research on Cancer, the global cancer incidence and mortality in the year 2012 were approximately 14.1 million (new cancer cases) and 8.2 million (deaths), respectively. The occurrence of cancer has been increasing due to risk factors such as smoking, obesity, physical inactivity, and changes in reproductive patterns related to urbanization, economic development, population growth, and aging^1^. Cancer treatment modalities include radiation therapy, surgery, chemotherapy, immunotherapy, and hormonal therapy. Approximately 50% of the cancer patients consider radiation therapy as an essential component of cancer treatment, and it contributes to 40% of the curative therapy for cancer^2^. RT is an indispensable method of cancer treatment in modern medicine. Stereotactic body radiotherapy (SBRT) is an emerging radiation treatment method that delivers a high dose of radiation to the target tissue and a minimum dose to normal tissues. It utilizes a single dose delivery or a small number of fractions with a high degree of precision within the body^3–8^.

Recently, clinical applications have been reported that use RT for the treatment of diseases other than cancer. For instance, the cardiac SBRT technique is a type of radiation therapy used for the treatment of ventricular tachycardia (VT)^9,10^. Several hospitals have reported that SBRT can reduce episodes of VT in cases where symptoms fail to improve after receiving conventional treatments, drugs, and electrode ceramics. RT in patients suffering with VT is aimed to eliminate the symptoms, and SBRT can achieve delivery of high dose and single separation for heart diseases. The method of SBRT in which a single prescribed dose of 25 Gy is delivered to a patient has emerged as a therapeutic tool to manage highly refractory VT^11^.

However, intensive care should be ensured for patients treated with an uncommon prescribed dose of 25 Gy. Excessive exposure to lung tissue can cause radiation-induced lung injury (RILI). RILI encompasses lung toxicity induced by RT that acutely manifests itself as radiation pneumonitis, bronchiolitis obliterans organizing pneumonia (BOOP), and chronically as radiation pulmonary fibrosis^12,13^.

Furthermore, previous studies have demonstrated different values and compared them with those of measured doses under field conditions for water-equivalent, lung, rib, and hard bone densities for dose calculation algorithms in the RTP system^14^. Additionally, the total dose calculated by the Acuros XB algorithm (AXB) was closer to the measured dose than that of the dose calculated by the anisotropic analytic algorithm (AAA) algorithm^15^. In particular, the similar results were observed in the phantom evaluation, and the calculation was different in the density-changing zones (substance boundaries) between AXB and AAA^16^.

Therefore, it is crucial to investigate the actual dose difference from the treatment planning dose distributions if the density of surrounding tissues is significantly different from that of the planning target volume (PTV), such as in a superficial cardiac lesion surrounded by low-density lungs.

The aim of this study is to evaluate the rebuild-up effect and dose discrepancy based on the measured dose distributions as compared to the calculated doses from AXB and AAA algorithms for superficial cardiac lesion surrounded by low-density lungs using a 3-D printed cardiac phantom.

## Materials and methods

### 3-D printed cardiac phantom

**Figure 1:**
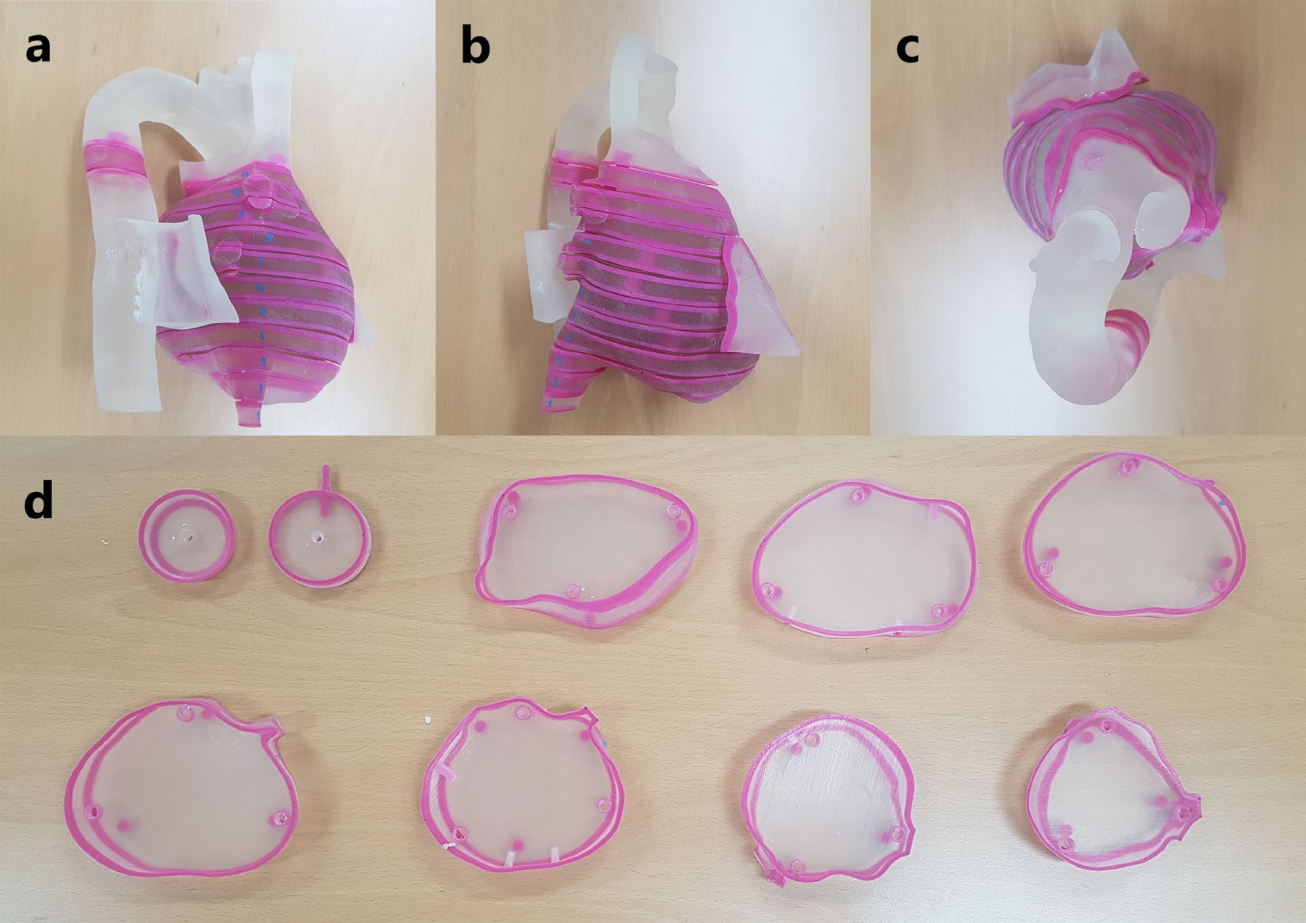
3-D printed cardiac phantom set. (**a**) Lateral view, (**b**) anterior view, (**c**) top-down view, and (**d**) cardiac phantom slabs.

We designed a 3-D printed cardiac phantom to emulate SBRT for VT treatment. The 3-D printed cardiac phantom was designed and printed referring to the humanoid Lungman (KYOTO KAGAKU, Japan) phantom CT images, which reproduced the pulmonary artery and the aortic arch. The size of the 3-D printed cardiac phantom was approximately 10 cm in diameter at the broadest center of the heart and an aortic arch of approximately 13 cm in width, 15 cm in length, and 17 cm in height. The 3-D design tool used a 3-Matic medical program (Mimics, Materialise, Belgium), and a 3-D printer (objet500 connex3, Stratasys, Eden Prairie, MN) was used to fabricate the phantom. It was designed to fix the Lungman phantom and resemble the human heart using Agilus and Magenta, which mimic human tissue (Fig. 1). Furthermore, the 3-D printed cardiac phantom was developed to insert Gafchromic EBT3 films (Ashland, Bridgewater, NJ), which can be separated into 11 slabs at 1-cm intervals, allowing the Gafchromic EBT3 film to settle in its location after insertion.

**Figure 2:**
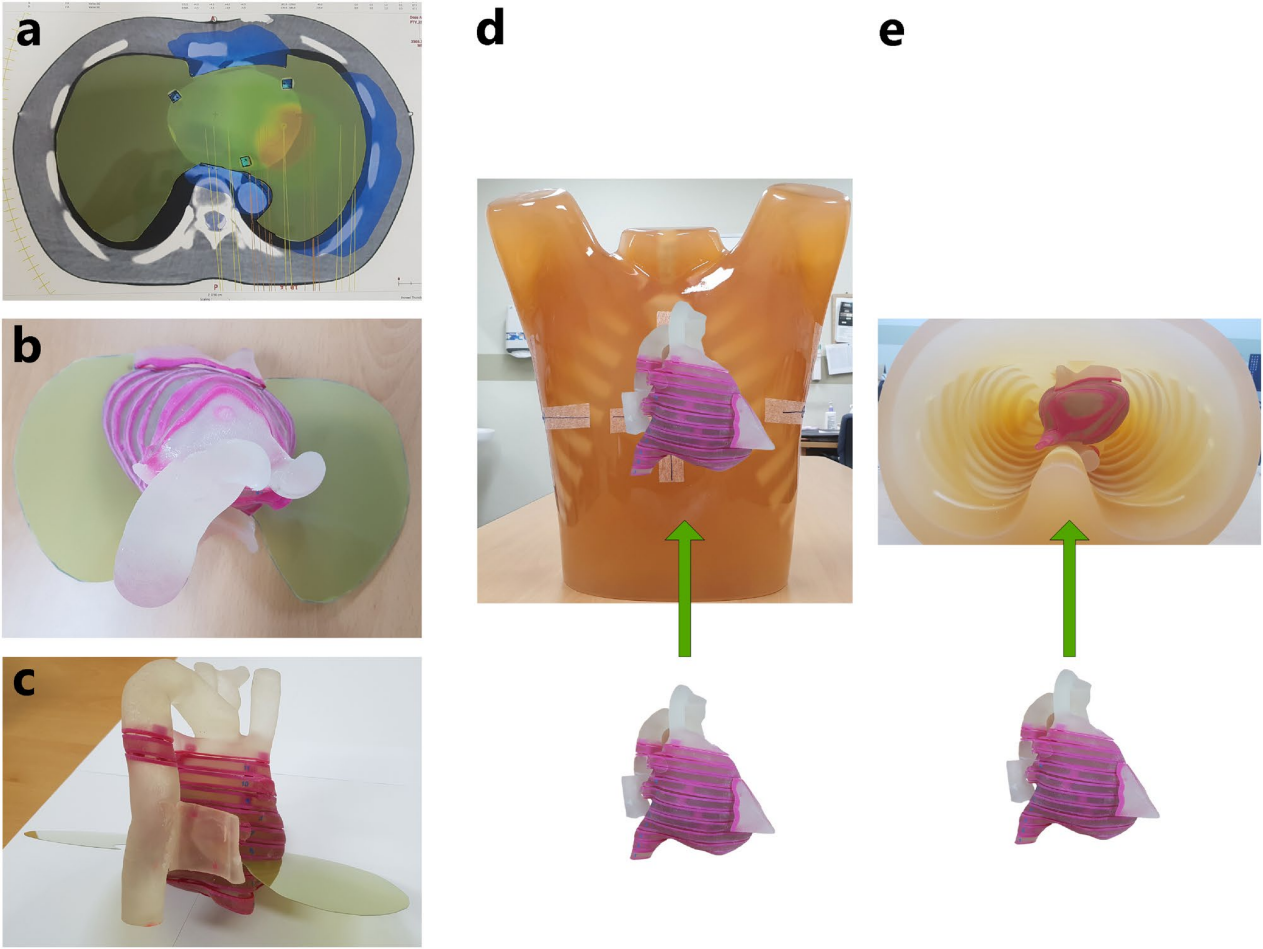
Film dosimetry set-up with 3D printed cardiac phantoms. (**a**) Axial view, (**b**) top-down view, (**c**) lateral view of the 3-D cardiac phantom, and (**d**) anterior-oblique view, (**e**) bottom-up view of the Lungman phantom inserted 3-D printed cardiac phantom.

The films were cut and inserted in the 3-D printed cardiac slabs. As shown in Fig. 2, the Gafchromic EBT3 film is ready for analysis across the entire heart and lungs, corresponding to the region of interest (ROI) (Fig. 2). The Lungman phantom was used, which is similar to that of the shape of a human chest, as shown in Fig. 2. The 3-D printed cardiac phantom with the Gafchromic EBT3 film were placed inside the Lungman phantom (Fig. 2). After inserting the 3-D printed cardiac phantom, the remaining space was filled inside the Lungman phantom with styrofoam grains, and the bottom cover of the Lungman phantom was closed to secure it.

### VMAT planning and film dosimetry

**Figure 3:**
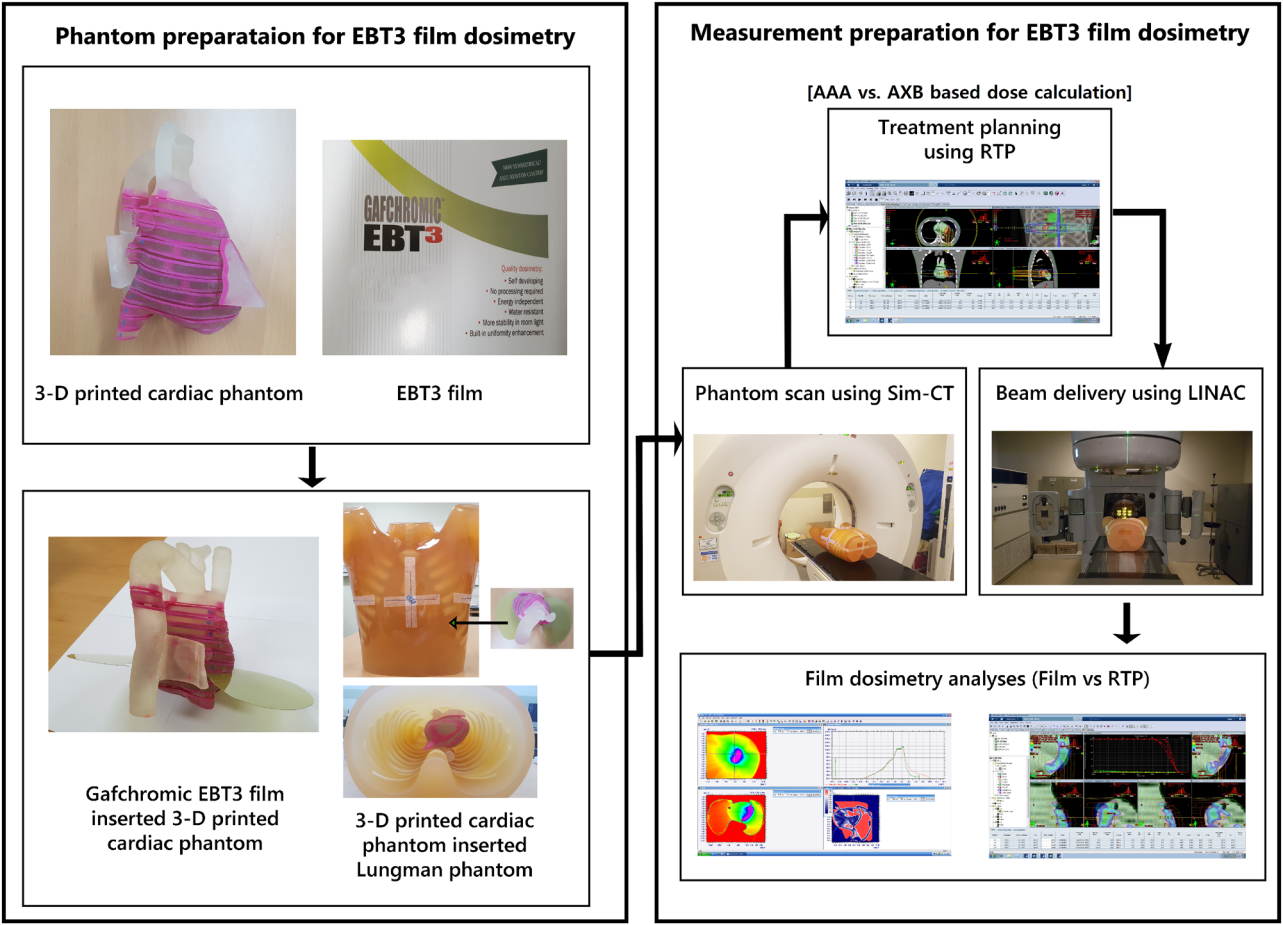
Overall procedures of the experiments and analysis for the study.

A CT image set of the Lungman phantom inserted with a 3-D cardiac phantom was obtained using a CT simulator (Large Bore, TOSHIBA, Japan). The prescribed dose scheme was 25 Gy in a single fraction for highly refractory ventricular tachycardia, aiming to eliminate symptoms of VT. The planning technique was adopted with four full (360°) or half (180°) arcs-based volumetric modulated arc therapy (VMAT) using 6-MV and 15-MV photon beams (Table 1). The dose-volume optimization parameters were similar for the experiments (PTV and GTV: 100, lt lung: 70, rt lung: 50, cord: 50, residual heart: 60 with 3–5 volume levels). Inverse planning for VMAT was applied to optimize the dose-volume basis using the AAA and AXB in a radiation treatment planning (RTP) system (Eclipse v. 13.6, Varian, Palo Alto, CA) (Table 1). We used 6-MV and 15-MV photon energies emitted from a LINAC (Clinac iX, Varian, Palo Alto, CA) to investigate dose rebuild-up effect and dose perturbation caused by different electronic disequilibrium. Film dosimetry scanners were used (DosimetryPRO Advantage Red, Vidar Systems Corporation, Herndon, VA) with a dedicated film dosimetry software (OP-IMRT, ver.1.6, IBA dosimetry, Germany). Finally, the measured dose was analyzed using film dosimetry and the measured dose distribution was compared to the planning dose distributions from the RTP system. Figure 3 shows the schematic flow chart of the entire process of dose verification (Fig. 3).

**Table 1:** Planning parameters volumetric modulation arc therapy (VMAT) plans.

Plan types	Energy	Arc types	Gantry angles (°)	Dose calculation algorithm
Plan 1/2	6-MV	Full	179.0–181.0	AAA/AXB
Plan 3/4	6-MV	Half	0.0–179.0	AAA/AXB
Plan 5/6	15-MV	Full	179.0–181.0	AAA/AXB
Plan 7/8	15-MV	Half	0.0–179.0	AAA/AXB

### Ethics approval and informed consent

Ethics approval and formal consent do not require a phantom study.

## Results

### Planning comparisons

**Figure 4:**
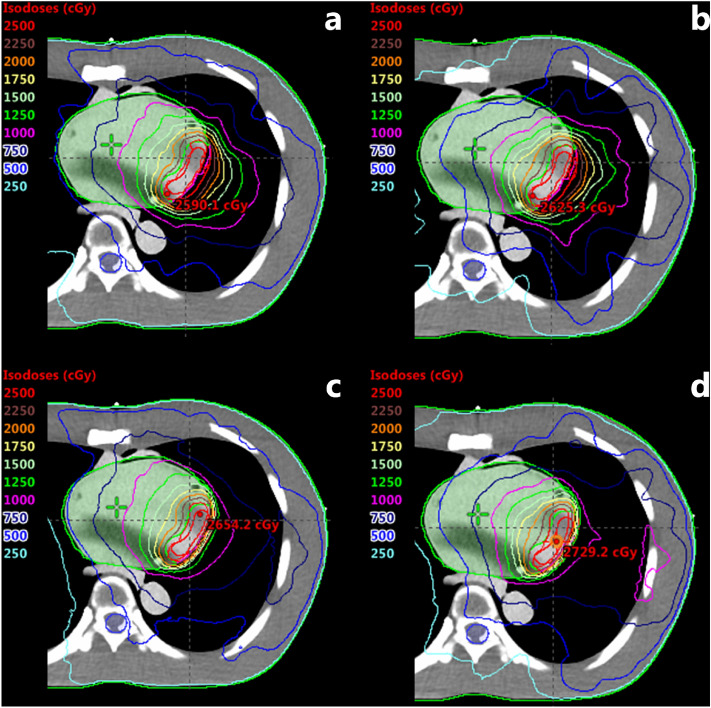
Volumetric modulated arc therapy (VMAT) plans using 6-MV photon beams. (**a**) Full arc treatment (AAA Algorithm), (**b**) half arc treatment (AAA Algorithm), (**c**) full arc treatment (AXB Algorithm), and (**d**) half arc treatment (AXB Algorithm).

**Figure 5:**
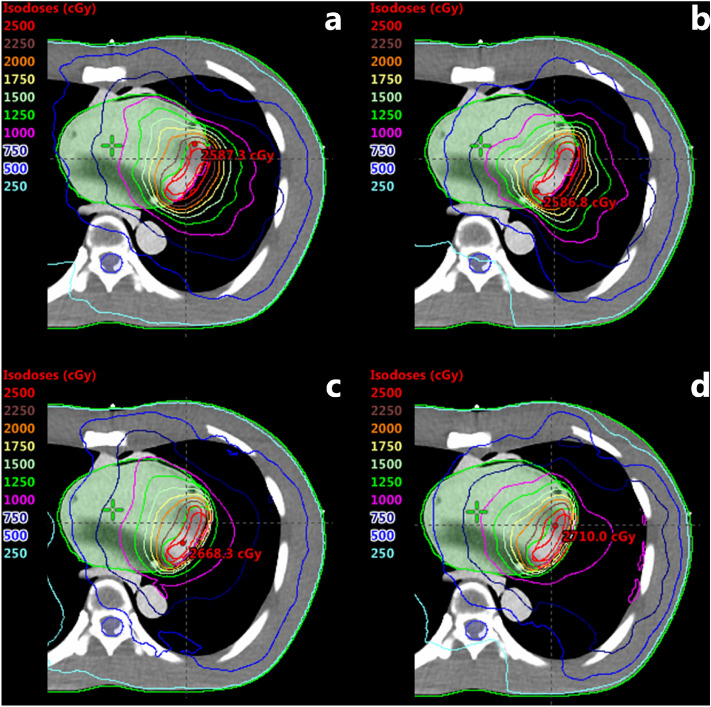
Volumetric modulated arc therapy (VMAT) plans using 15-MV photon beams. (**a**) Full arc treatment (AAA Algorithm), (**b**) half arc treatment (AAA Algorithm), (**c**) full arc treatment (AXB Algorithm), and (**d**) half arc treatment (AXB Algorithm).

**Figure 6:**
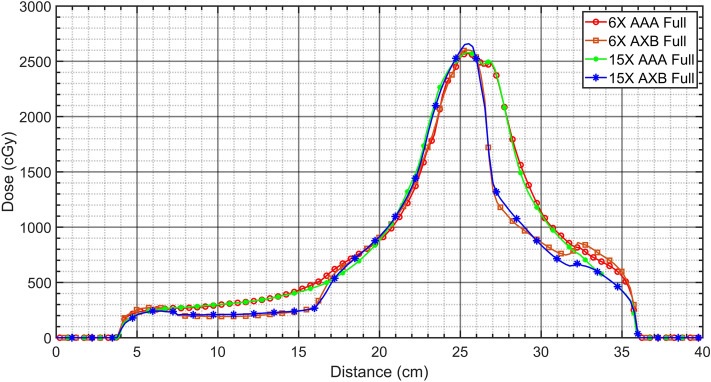
Comparison of dose profiles at the center of the PTV in the full arcs-based VMAT plans with different energies and caluation algorithms.

**Figure 7:**
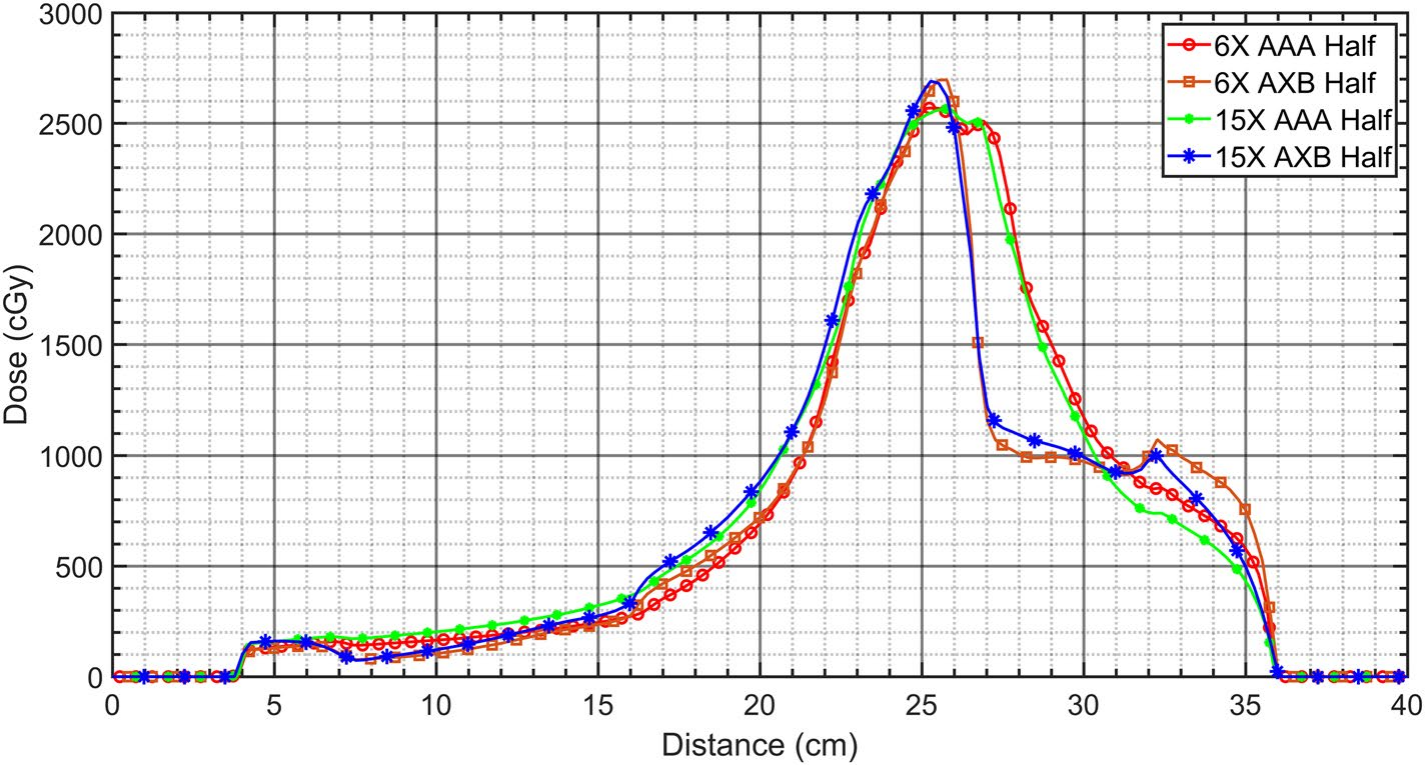
Comparison of dose profiles at the center of the PTV in the half arcs-based VMAT plans with different energies and caluation algorithms.

The VMAT plans demonstrated relatively good coverages and lower hot spot dose around the cardiac PTV for the AAA algorithm compared to that of the AXB-based VMAT plans, irrespective of energies and beam arc angles (Figs. 4 and 5). As shown in Figs. 6 and 7, the line profiles across cardiac PTV exhibit relatively poor coverages to the PTV in the AXB-based plans.

The differences were greater than 2 cm at the interfaces with lung and the cardiac PTV. In the case of only the VMAT plan, it was observed that the AXB algorithm considered the rebuild-up effect on the interface between low-density lung matter and the cardiac PTV surface.

**Figure 8:**
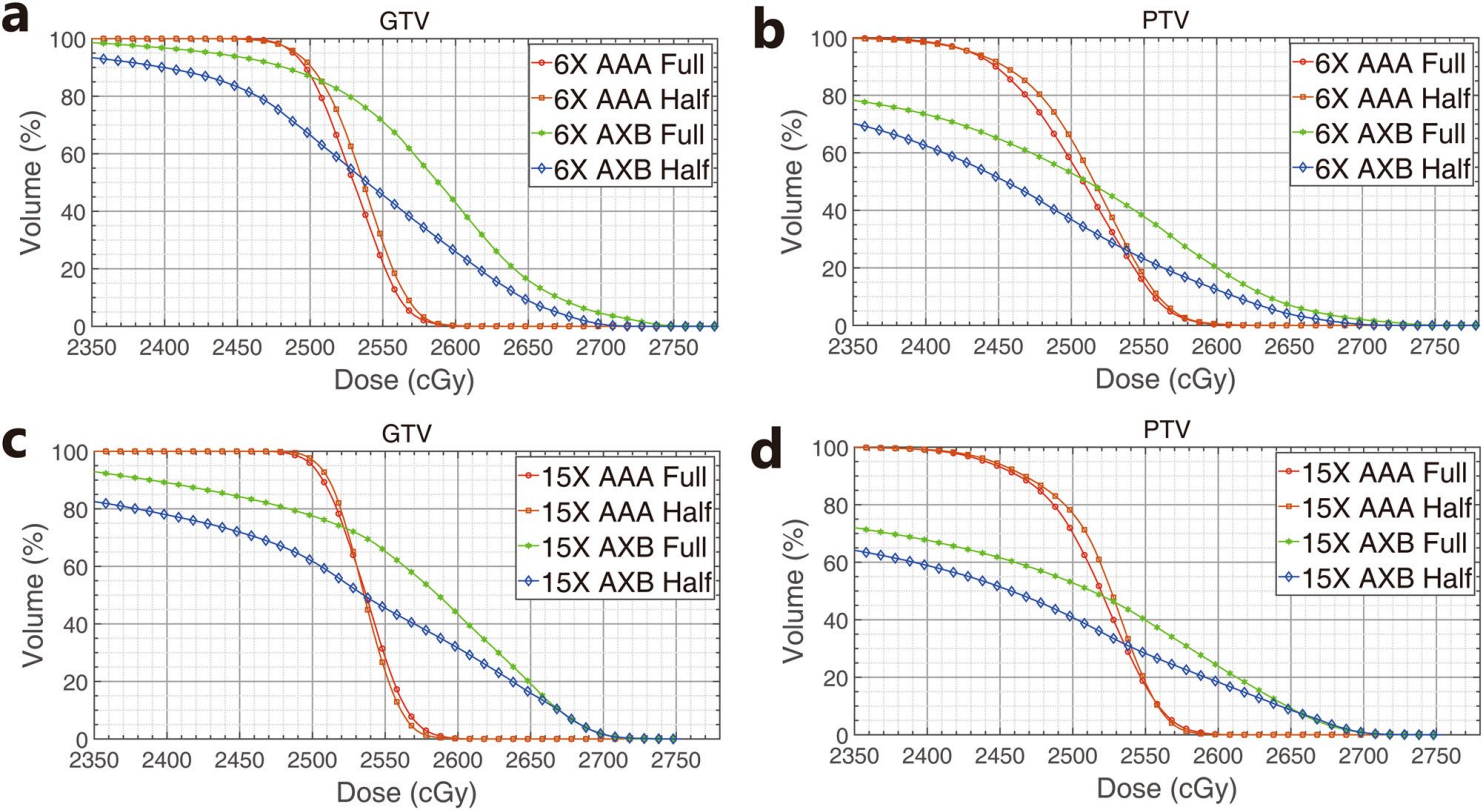
Dose-volume histograms for (**a**) GTV_p_ in the 6-MV plans, (**b**) PTV_25 in the 6-MV plans, (**c**) GTV_p_ in the 15-MV plans, and (**d**) PTV_25 in the 15-MV plans.

Figure 8 shows the DVH of GTV and PTV in the treatment plans according to calculation algorithms, energy, and arc angles. DVH analysis demonstrated that the dose coverages calculated using the AXB algorithm exhibited under-dose patterns compared to those of AAA. In addition, this phenomena were observed at 15-MV beam and at half arcs plans than that at 6-MV beam and full arcs plans.

Tables 2 and 3 show the dose statistics of the targets according to each treatment plan. In the case of full arcs plans, according to the calculation algorithm (AAA vs. AXB), V_95_ of PTV differed by 27% for 6-MV and 29% for 15-MV, and D_95_ for 6-MV and 15-MV demonstrated a difference of 24% and 30%, respectively. The maximum doses of the PTV in the AXB plans were 5.1% higher than those in AAA plans at 6-MV, and 3.8% higher at 15-MV. In addition, half arcs treatment plans showed a similar tendency to that of full arcs plans.

**Table 2:** Dose statistics of targets (GTV and PTV) from full arcs volumetric modulated arc therapy (VMAT) plans.

Energy	Calculation model	Arc	Structure	Volume (cm^3^)	D_95_ (%)	V_95_ (%)	Min dose (cGy)	Max dose (cGy)	Mean dose (cGy)
6-MV	AAA	Full	GTV	19.4	100.0	99.5	2446.0	2645.4	2529.6
			PTV	44.1	99.6	97.2	2241.4	2645.4	2504.8
6-MV	AXB	Full	GTV	19.4	97.8	97.3	2185.8	2780.7	2580.2
			PTV	44.1	76.1	70.6	1295.4	2780.7	2423.4
15-MV	AAA	Full	GTV	19.4	100.0	100.0	2438.8	2615.3	2536.5
			PTV	44.1	99.7	97.6	2244.0	2629.1	2514.6
15-MV	AXB	Full	GTV	19.4	91.1	92.6	2062.3	2727.7	2558.3
			PTV	44.1	69.9	69.0	1338.6	2727.7	2400.8

*GTV* gross tumor volume, *PTV* planning target volume.

**Table 3:** Dose statistics of targets (GTVp and PTV_25) from half arcs volumetric modulated arc therapy (VMAT) plans.

Energy	Calculation model	Arc	Structure	Volume (cm^3^)	D_95_ (%)	V_95_ (%)	Min dose (cGy)	Max dose (cGy)	Mean dose (cGy)
6-MV	AAA	Half	GTV	19.4	100.0	99.6	2416.5	2626.7	2534.9
			PTV	44.1	99.4	97.3	2266.0	2656.9	2510.7
6-MV	AXB	Half	GTV	19.4	91.9	92.7	2011.0	2729.2	2528.8
			PTV	44.1	66.8	61.3	1098.4	2729.2	2339.9
15-MV	AAA	Half	GTV	19.4	100.0	100.2	2436.9	2621.6	2535.8
			PTV	44.1	99.7	97.8	2259.0	2628.6	2519.3
15-MV	AXB	Half	GTV	19.4	80.4	85.8	1808.7	2754.5	2498.4
			PTV	44.1	61.7	58.7	1050.1	2754.5	2308.5

### Verification using film dosimetry

**Figure 9:**
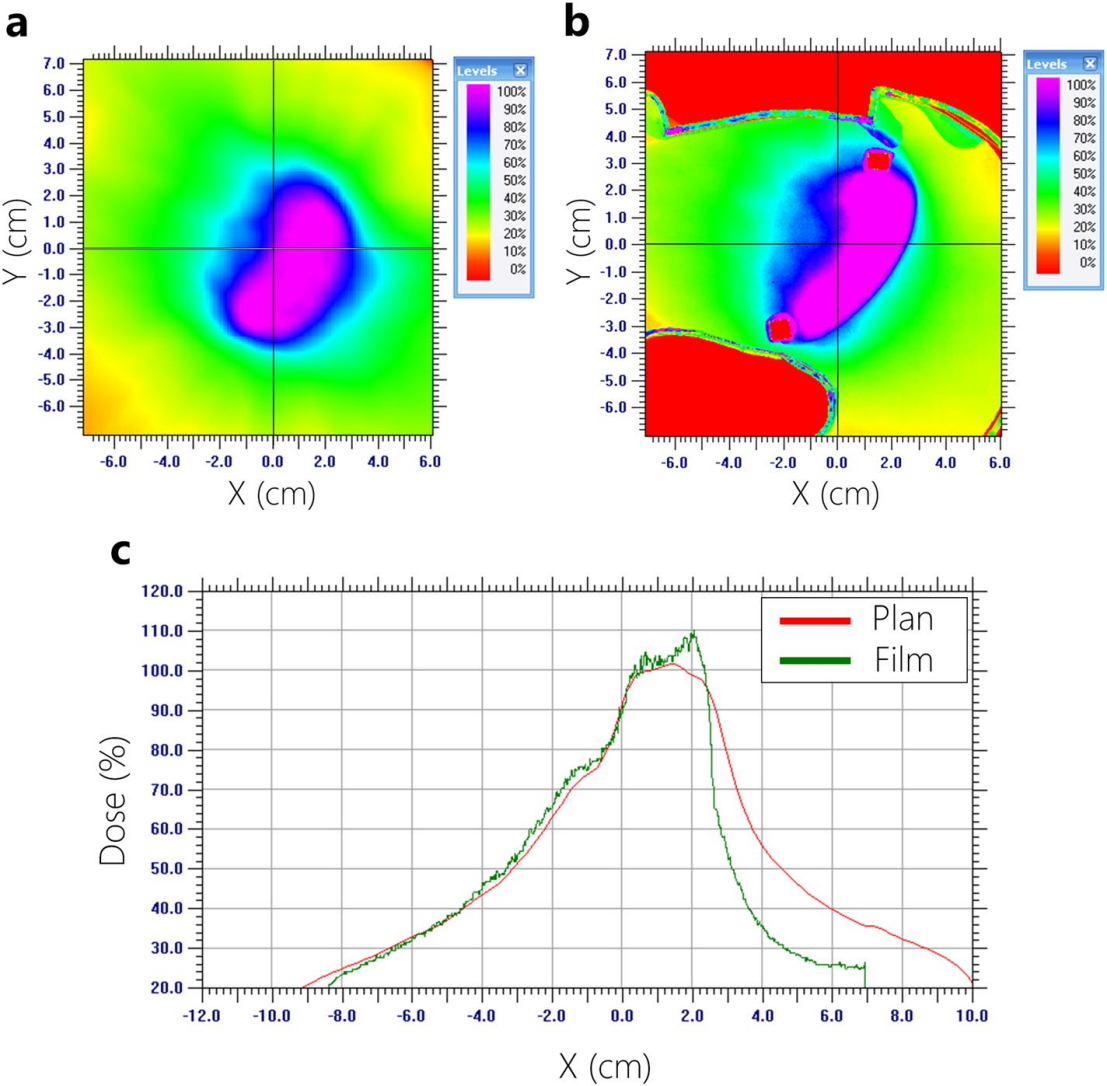
Dose profile analysis (measured film vs. planned dose) using 6-MV based on AAA Algorithm. (**a**) Planned dose, (**b**) measured dose, (**c**) comparison of dose profiles.

**Figure 10:**
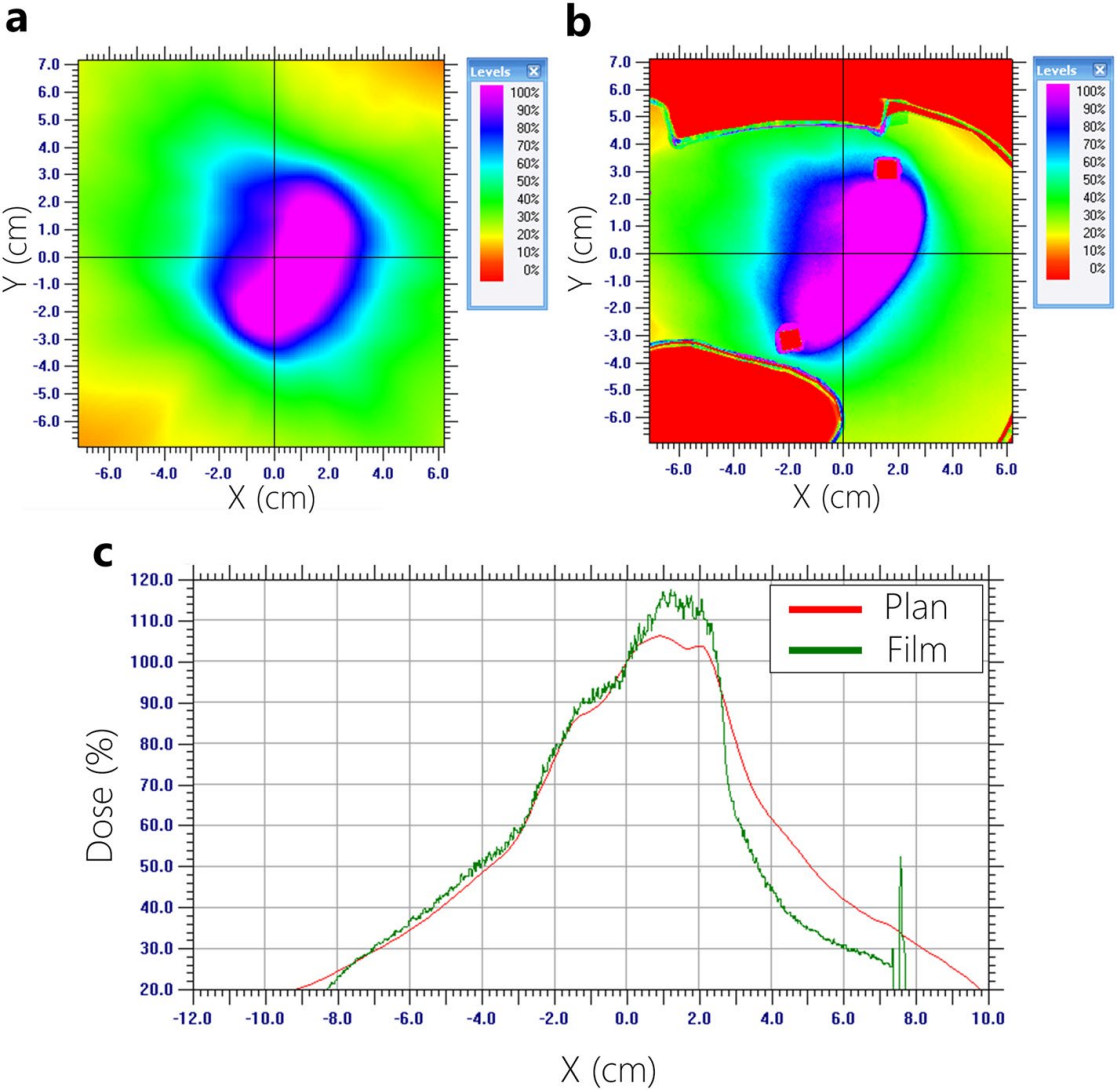
Dose profile analysis (measured film vs. planned dose) using 15-MV based on AAA Algorithm. (**a**) Planned dose, (**b**) measured dose, (**c**) comparison of dose profiles.

**Figure 11:**
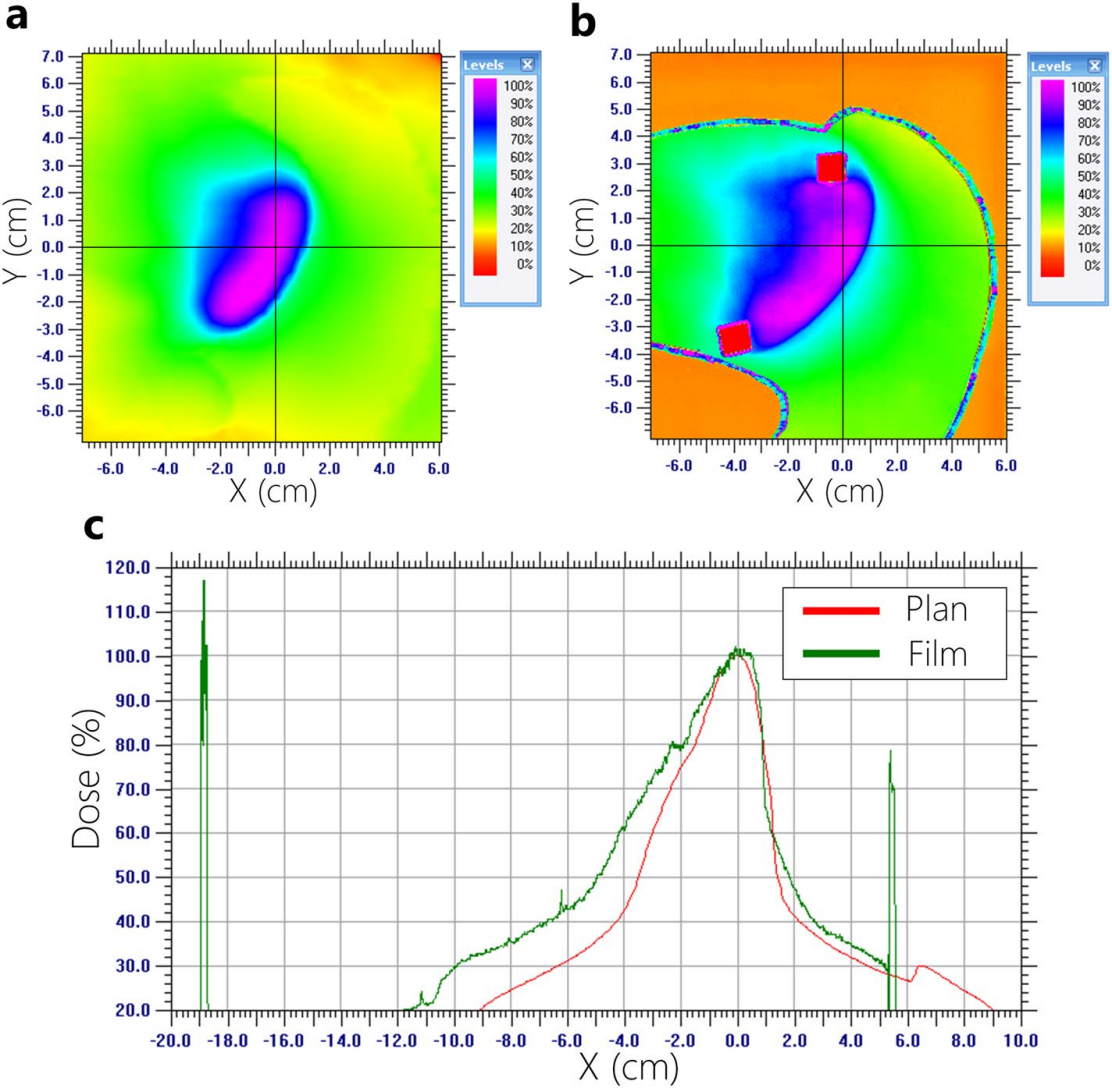
Dose profile analysis (measured film vs. planned dose) using 6-MV based on AXB Algorithm. (**a**) planned dose, (**b**) measured dose, (**c**) comparison of dose profiles.

**Figure 12:**
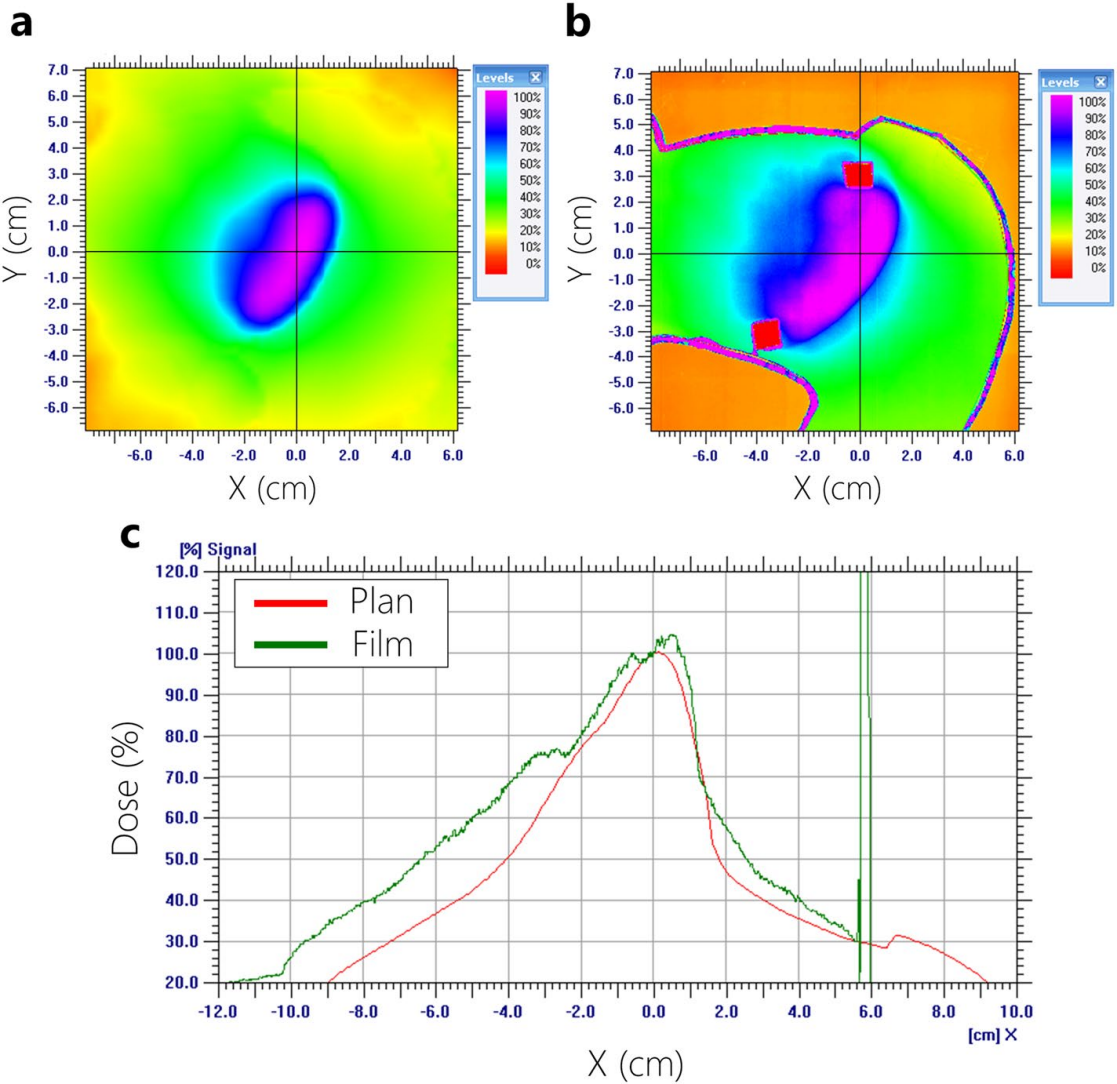
Dose profile analysis (measured film vs. planned dose) using 15-MV based on AXB Algorithm. (**a**) planned dose, (**b**) measured dose, (**c**) comparison of dose profiles.

Figures 9, 10, 11 and 12 show the results of quantitative comparison of the film measurements according to the planned irradiation condition with the calculated dose distributions from the VMAT plans using the 3-D printing cardiac phantom.

Film dosimetry showed a significant difference from the planned results in the AAA plans. Particularly, the dose mismatch occurred between the cardiac PTV and the left lung interface. In the case of 6-MV plans calculated by AAA, the maximum dose increased from 4.1 to 7.7% on the PTV. Furthermore, it showed that 50% of the width of dose profiles was reduced by 1.3 cm in the 6-MV plan (Fig. 9). Figure 10 shows that 15-MV beam energy also revealed a dose mismatch between the cardiac PTV and the left lung interface. The maximum dose increased from 4.1 to 8.1% in the PTV, indicating that 50% of the width of the dose profiles was reduced by 2.3 cm in the interface of the cardiac PTV and the left lung (Fig. 10). Conversely, in the case of the plans using the AXB algorithm, the maximum dose increased by 2.0–5.0%. In contrast to the AAA algorithm, the dose patterns at the interface demonstrated a good agreement with the plans. However, a dose increment pattern was observed in the medial side of the PTV (Figs. 11 and 12). Additionally, it was observed that 50% of the width of the dose profiles were increased by 1.2 cm and 2.4 cm on 6-MV and 15-MV photon beams, respectively.

## Discussion

Undesirable errors are encountered due to unknown reasons related to various dosimetric factors such as beam energies, delivery methods, and dose calculation algorithms while optimizing the dose distributions in commercial RTP systems. Uncommon cases such as superficial cardiac lesion adjacent to low-density lung tissue might be susceptible to such errors. This is because the low-density portion might affect an accurate calculation of dose distribution due to complex physical interactions such as rebuild-up effect. There might be two main factors in the case of superficial cardiac lesions surrounded by low-density lung tissues. One is the forward-directed photon fluences due to the decreased attenuation of the beam intensity, while the other is the effect of electronic disequilibrium where the low-density lung tissue intersects the cardiac lesion with a generic density. Dose rebuild-up effect might occur, which results in the delivery of an unwanted lower dose to the superficial cardiac lesion. These two factors are individually affected due to varying densities, with dominant factors depending upon beam energy, field size, and local inhomogeneity distributions^17^.

The results of this study are significant because this type of dose verification can be a difficult trial with a well-emulated humanoid phantom. Additionally, design and fabrication of the inserted cardiac phantom is a delicate process to ensure precise film dosimetry. It is beneficial to utilize 3-D printing technology for this specialized dosimetry purpose. Film dosimetry with a dedicated humanoid phantom enabled the determination of dose mismatch between the cardiac PTV and the left lung interface. The International Commission on Radiation Units and Measurements (ICRU) recommends an overall accuracy limit of ± 5% for dose delivery^18^, stating that it is crucial to maintain density-related dose uncertainty within a small range if it is technically achievable. The ICRU recommends setting the dose uncertainty to the smallest for tumors while maintaining steep dose–response curves and narrow therapeutic windows.

Inhomogeneity correction is performed by photon dose calculations considering the mass density or electron density information derived from CT-density conversion tables in an RTP system. Inaccurate density information can cause dose errors when non-uniformity correction is applied, and several studies have proposed tolerance levels to maintain dose errors within an acceptable limit^19,20^. However, extensive changes in relative electron density, such as in the lungs, soft palate, and bones, are significantly more expansive than the recommended tolerance values for CT number accuracy testing of diagnostic CT scanners. For this reason, the resulting dose error of the target volume will be < 2% for most clinical cases and < 3% for challenging lung SBRT cases if the changes in density are limited to ± 0.02, ± 0.03, and ± 0.10 g/cm^3^ for the lungs, soft tissue, and bones, respectively^17^. In addition, it was observed that specific irradiation conditions should be considered such as geometrical factors of inhomogeneous medium, along with the application of beam energies and appropriate CT-density calibration.

Additionally, dose errors can be reduced depending upon the selection of the dose calculation algorithm.

Previous studies have demonstrated that the difference between AAA and AXB in radiotherapy plans for lung cancer is less than 3% on PTV^21^. Furthermore, a comparison between AAA and AXB in individual IMRT fields demonstrated that the dose differences for a single beam might be up to 8% in the lung interface regions, even if the total dose differences are minor. If the PTV is close to the interface region, especially when the bone tissue is involved, the differences between AAA and AXB might be significantly large^22^.

It was observed that film dosimetry with 3-D printed phantom can be a feasible tool to perform challengeable dosimetry, such as dose discrepancies of high gradient dose interface in a humanoid structure.

Dose fluctuation on the interface between superficial cardiac lesions and low-density lungs can lead to an error in the estimation of accurate dose delivery for the case of VT SBRT. Additionally, 3-D printing techniques can be a feasible dosimetry tool for the verification of dose discrepancies.

## Data Availability

The datasets used and analyzed during the current study are available upon reasonable request. Please contact the authors for data requests.
